# Top five unanswered questions in fungal cell surface research

**DOI:** 10.1016/j.tcsw.2023.100114

**Published:** 2023-11-03

**Authors:** Neil A.R. Gow, Arturo Casadevall, Wenxia Fang

**Affiliations:** a Medical Research Council Centre for Medical Mycology, University of Exeter, Geoffrey Pope Building, Stocker Road, Exeter EX4 4QD, United Kingdom; b School of Medicine, Department of Molecular Microbiology and Immunology, Johns Hopkins, Bloomberg School of Public Health, 615 N. Wolfe Street, Baltimore, MD 21205, USA; c Institute of Biological Sciences and Technology, Guangxi Academy of Sciences, Nanning, Guangxi, China

**Keywords:** Drug target, Permeability, Stress, Fungal vaccine

## Introduction

We sent requests to authors of manuscripts published in The Cell Surface and community researchers working on fungal cell walls to send notes about what they considered were the top five unanswered questions in the field of fungal cell wall and cell surface biology. This article summarizes the feedback that was received by them as well as the views of the authors. In this issue you can find equivalent syntheses for researchers working on bacterial, unicellular parasite and plant systems.

## Unanswered question #1 – How do fungal walls adapt to changing environments and imposed stresses by reorganising their cell walls?

Cell wall structures are normally determined for cells taken from cultures harvested from one set of environmental conditions. We now know that fungal walls are exquisitely sensitive to changes in growth conditions (carbon and nitrogen source, pH, temperature, osmotic pressure) and that they regulate cell wall structure an composition in response to a wide range of environmental stresses (oxidative, nitrosative, antifungal and immune stress) (Pradhan et al., 2019, Gow and Lenardon, 2023). Cellular responses to environmental insults that threaten the integrity of the cell wall responses have to be rapid to avoid irreversible loss of viability. These responses involve complex transcriptional and post-transcriptional regulatory networks and epigenetic changes that result in architectural modifications to how the wall is assembled, disassembled and the component parts are attached to each other. At present we have only evolved a rudimentary understanding of some of sensors of cell wall stress, the signalling pathways and the resultant cell wall modifications. Essential information is lacking about how these circuits are integrated and how the temporal stress-to-response pathway is articulated to preserve wall integrity and essential cell wall functions in 3-D space. To drive progress in this field it will be necessary to understand with precision how tension in the cell wall is measured, how chitin and β-1,3 glucan cross-linking is regulated to create dynamic tension in the wall, and how cell wall proteins and other carbohydrates and wall components (β-1,6-glucan, galactomannan, galactosaminoglycan, melanin, hydrophobins etc), are coupled to, and decoupled from the chitin-glucan skeleton (Levin, 2005, Clavaud et al., 2012). In a dynamic wall that is constantly being remodelled, how is assembly, disassembly and reassembly of its components regulated without dangerously threatening the integrity of the cell? And how do these mechanisms respond to changing *in vivo* conditions and the application of drugs such as fosmanogepix, ibrexafungerp and echinocandins that damage the cell wall?

## Unanswered question #2 – How is cell wall rigidity and permeability controlled?

In addressing the question of cell wall rigidity, one must first discard the wall metaphor, which limits the imagination and creates false notions of microscopic reality (Casadevall and Gow, 2022). Instead of a wall, the outer fungal cell structure is more like a flexible mesh (Casadevall and Gow, 2022) that is rapidly remodeled during cell elongation, replication, dimorphic transitions etc. Perhaps the closest analogy from our macroscopic world would be to view the fungal cell wall like medieval chain mail, which could provide protection while being flexible and taking the shape of the wearer. However, even that image is incomplete since the fungal cell wall can allow the passage of extracellular vesicles and even bacteria, which would be akin to mesh armour allowing the passage of apples and soccer balls. In fact, the cell wall allows bidirectional passage of vesicles such that export structures can pass to the exterior while liposomes containing amphotericin B can enter from the outside (Walker et al., 2018). The mechanisms for cell wall transit are unknown but these must involve rapid rearrangement of its intricate polysaccharide components or perhaps the existence of pores of varying diameter that allow macromolecular structures, possibly with the aid of molecular motors. To understand how diffusion of nutrients and drugs is regulated one must have more information on the porosity of the cell wall structure, including its permeability to molecules of different size, charge, and hydrophobicity, and we must discriminate between passive and active processes. Such studies would be greatly aided by experimental ability to construct cell wall structures in vitro – something that is currently not possible. For melanized cell walls the problem is further complicated by the fact that any cell wall rearrangement involving remodeling of the melanin layer, affects permeability (Eisenman et al.,2005) and is therefore likely to interfere with inward vesicular penetration (Walker et al., 2018).

## Unanswered question #3 – How can cell wall polysaccharide synthesis be targeted by antifungals and vaccines?

Considering the cell wall of fungi is made exclusively from molecules that are not represented in the human body, it is surprising how little cell wall biology has been exploited to date as drug and vaccine targets. Glycoconjugates are major targets of vaccines against other pathogen groups, but have yet to be exploited in medical mycology. But some fungal diseases are due to failure of the immune system to sufficiently activate immunity whilst others are due to uncontrolled inflammation triggered by fungal cell wall recognition (Lionakis et al., 2023). It will be important to learn what features of cell wall epitopes make them potent agonists and antagonists of immune recognition. To date there are no fungal vaccines. Why not? The Als3 GPI-linked cell wall protein of *C. albicans* GPI-anchored showed efficacy in a phase 2 clinical trial (Edwards et al., 2018), but it may be that polyvalent vaccines are required for optimal protection. In terms of antifungal drugs echinocandins and ibrexafungerp both target biosynthesis of β-(1,3)-D-glucan in the fungal cell wall and fosmanogepix targets the inositol acyltransferase Gwt1 that is essential for (GPI) anchor biosynthesis and hence the coupling of essential cell wall proteins to the carbohydrate wall skeleton. We still lack details about the modes of action of these agents and whether the efficacy and spectrum of activity of these drugs can be potentiated in combinatorial formats (Perfect, 2017). Also might inhibitors that compromise cell wall salvage pathways such as the calcineurin pathway that are induced in response to antifungal therapy act synergistically with the primary antifungal drug? (Steinbach et al., 2007). A combination of solid-state NMR (Chakraborty et al., 2021, Fernando et al., 2022) to understand cell wall architecture, cryo-TEM and structural biology to resolve structure–function relationships of the key wall biosynthesis enzymes, combined with new computational, data sciences and AI approaches may speed future progress to a more rational approach to drug discovery.

## Unanswered question #4 – What roles do specific polysaccharides play in assembly, deposition, and immune interactions?

A magnificent spectacle in the fungal world is the change in cell shape that is triggered by cellular stress. Examples of this are the yeast-hypha transition in the dimorphic fungi and the phenomenon of cell gigantism (Titan cells, Goliath cells, spherules) or dwarfism observed for *C. neoformans* (Gow and Lenardon, 2023). Equally remarkable is that such changes occur very rapidly with germ tube formation in *C. albicans*, occurring within 30 min after exposure to mammalian serum. Changes in cellular shape are accompanied by changes in cell wall composition, volume, and the distribution of organelles. For the cell wall this implies that there must be rapid rearrangements of its components, which inevitably implies that some components are removed while others added in an orderly way with the formation of new chemical linkages. Furthermore, since it is difficult to imagine that the cell has an architectural map to direct the remodeling of the cell wall or a mechanism of central planning, it is likely that the cellular transition is the result of local rules resulting from component interactions from which the final form emerges. In chemistry and biology complex structures can emerge from components that follow simple rules, with examples being the formation of crystals from a few atomic interactions and the self-organization of DNA into fractal-like Sierpinski triangles (Rothemund et al., 2004). If this is the case, then much of the information for the formation of hyphal, giant and yeast cells must be contained in the individual cell wall molecules with the final cellular form emerging from the myriad of component interaction that occur between them. If the process of cell wall reorganization emerges from a complex choreography of its components, then understanding the mechanism would require both reductionistic and holistic approaches, with the former involving precise temporal mapping of cell wall components combined with chemical determination of their possible interactions and the latter mathematical modeling to illustrate how shape complexity and flexibility emerges from such interactions.

## Unanswered question #5 – How does the fungal cell wall interact with environmental organisms and molecules?

The intricate interplay between fungal cell walls and the environment plays a pivotal role in the context of antifungal immunity. It commences with the critical recognition of specific pathogen-associated molecular patterns (PAMPs) on the fungal cell surface. Yet, fungal pathogens demonstrate remarkable adaptability, enabling them to conceal or even enzymatically remove of modify these PAMPs, particularly under specific environmental conditions such as iron limitation. This prompts the activation of non-canonical signaling pathways for immune evasion (Pradhan et al., 2019). The mechanisms employed by fungal pathogens to mask or modify PAMPs and any emerging host recognition strategies continue to be active areas of research and was highlighted as a key research question by several investigators responding to our questionnaire. In addition to their role in antifungal immunity, fungal cell walls are instrumental in shaping microbial relationships and community functions. For example, the secretion of cell wall polysaccharides or proteinase into the biofilm matrix enhances *Staphylococcus aureus* tolerance to antimicrobials and promoting adhesion between *C. albicans* and bacteria like *Enterococcus faecalis* and *Streptococci*. These interactions are crucial in biofilm development (Nogueira et al.,2019). Innovations, including biomarkers and therapies targeting microbial metabolites, hold promise for enhancing treatment efficacy in bacterial-fungal polymicrobial infections. In the realm of plant-fungal interactions, recent research has shed light on the intriguing role of an *exo*-β-1,3-glucanase known as Ebg1. This enzyme has been found to suppress β-1,3-glucan-triggered plant immunity by breaking down β-1,3-glucan and laminarin into glucose (Liu et al., 2023). However, what implications might this discovery have for plant-fungal interactions? Does Ebg1′s function as a PAMP imply the existence of yet-to-be-discovered cell wall-associated PAMPs, and how might this impact our understanding of plant defence mechanisms in the face of fungal pathogens? Fungal cell walls' interface with environmental chemicals and enzymes prompts key questions. How can we use this knowledge to enhance fungicides and pest control? What practical uses exist for enzymes from marine fungi in industry and the environment? Furthermore, how do fungal cell walls impact ecosystem dynamics, including biodiversity and nutrient cycling?

## CRediT authorship contribution statement

**Neil A.R. Gow:** Writing – review & editing, Writing – original draft, Validation, Funding acquisition, Formal analysis, Data curation, Conceptualization. **Arturo Casadevall:** Writing – review & editing, Writing – original draft, Validation, Investigation, Funding acquisition, Formal analysis, Data curation, Conceptualization. **Wenxia Fang:** Writing – review & editing, Writing – original draft, Validation, Project administration, Funding acquisition, Formal analysis, Data curation, Conceptualization.

## Declaration of Competing Interest

The authors declare that they have no known competing financial interests or personal relationships that could have appeared to influence the work reported in this paper.
